# EuPaGDT: a web tool tailored to design CRISPR guide RNAs for eukaryotic pathogens

**DOI:** 10.1099/mgen.0.000033

**Published:** 2015-10-30

**Authors:** Duo Peng, Rick Tarleton

**Affiliations:** ^1^​ Center for Tropical and Emerging Global Diseases, University of Georgia, Athens, GA 30602, USA; ^2^​ Department of Cellular Biology, University of Georgia, Athens, GA 30602, USA; ^3^​ Institute of Bioinformatics, University of Georgia, Athens, GA 30602, USA

**Keywords:** CRISPR-Cas9, eukaryotic pathogens, genome editing, gRNA design, webserver

## Abstract

Recent development of CRISPR-Cas9 genome editing has enabled highly efficient and versatile manipulation of a variety of organisms and adaptation of the CRISPR-Cas9 system to eukaryotic pathogens has opened new avenues for studying these otherwise hard to manipulate organisms. Here we describe a webtool, Eukaryotic Pathogen gRNA Design Tool (EuPaGDT; available at http://grna.ctegd.uga.edu), which identifies guide RNA (gRNA) in input gene(s) to guide users in arriving at well-informed and appropriate gRNA design for many eukaryotic pathogens. Flexibility in gRNA design, accommodating unique eukaryotic pathogen (gene and genome) attributes and high-throughput gRNA design are the main features that distinguish EuPaGDT from other gRNA design tools. In addition to employing an array of known principles to score and rank gRNAs, EuPaGDT implements an effective on-target search algorithm to identify gRNA targeting multi-gene families, which are highly represented in these pathogens and play important roles in host–pathogen interactions. EuPaGDT also identifies and scores microhomology sequences flanking each gRNA targeted cut-site; these sites are often essential for the microhomology-mediated end joining process used for double-stranded break repair in these organisms. EuPaGDT also assists users in designing single-stranded oligonucleotides for homology directed repair. In batch processing mode, EuPaGDT is able to process genome-scale sequences, enabling preparation of gRNA libraries for large-scale screening projects.

## Impact Statement

Like other communities, the eukaryotic pathogen research community has begun to adopt the CRISPR-Cas9 system for engineering genomes of eukaryotic parasites at an unprecedented scale and ease. Our web-based Eukaryotic Pathogen gRNA Design Tool (EuPaGDT) enables researchers to arrive at well-informed guide RNA (gRNA) and homology-directed repair template design for CRISPR-Cas9 experiments in eukaryotic pathogens. Using an array of known principles, EuPaGDT characterizes all potential gRNAs in a user-input gene, allowing users to quickly identify refined gRNA in a gene sequence and greatly increases the chances of successful CRISPR-Cas9 experiments. EuPaGDT also features a batch processing mode, which can identify gRNA at a whole genome scale, allowing the *in silico* preparation of gRNA libraries for large-scale screening projects. EuPaGDT is currently available for 25 eukaryotic pathogen genomes, covering major lineages of eukaryotic pathogens and popular model organisms. Users can also upload custom genomes or request default genomes to be added to EuPaGDT. At this time, the EuPaGDT server has been running for 5 months, and has processed over 1340 requests from 654 users originating from 30 countries.

## Introduction

RNA-guided Cas9 nuclease has enabled rapid, targeted modification of a wide range of genomes, including those of eukaryotic pathogens (Peng *et al.*, 2015; Shen *et al.*, 2014; Sidik *et al.*, 2014; Sollelis *et al.*, 2015; Zheng *et al.*, 2014), which are the causative agents of some of the most devastating and intractable diseases of humans. The CRISPR (clustered regularly interspaced short palindromic repeats)-Cas9 system is likely to be a particularly important tool for the study of gene function in pathogens that lack functional RNAi pathways, such as *Plasmodium* sp. (Ghorbal *et al.*, 2014; Lee & Fidock, 2014; Wagner *et al.*, 2014; Zhang *et al.*, 2014), the causative agent of malaria, and *Trypanosoma cruzi*, the agent of Chagas disease (Peng *et al.*, 2015). The CRISPR-Cas9 system has proven especially useful because of its relative ease of use and high efficiency as well as the ability to achieve multiple modifications per cell in a single organism. This latter property is particularly useful for modifying members of multigene families that are common in these pathogens (Peng *et al.*, 2015).

Current guide RNA (gRNA) design tools for the CRISPR-Cas9 system have limitations when applied to gRNA design for eukaryotic pathogens. For example, the genomes of most parasites exhibit great nucleotide sequence divergence (even at the within-species level) and harbour large, rapidly evolving gene families that are important players of host–pathogen interactions. To harness CRISPR-Cas9’s multiplexing power to edit gene families, a gRNA design tool must handle multiple ‘on-target’ hits (gene family members) and discriminate them from true off-target sequences. To address these problems, we have developed a web tool, Eukaryotic Pathogen gRNA Design Tool (EuPaGDT), tailored to design gRNA for eukaryotic pathogens.

## EuPaGDT

**Fig. 1.**
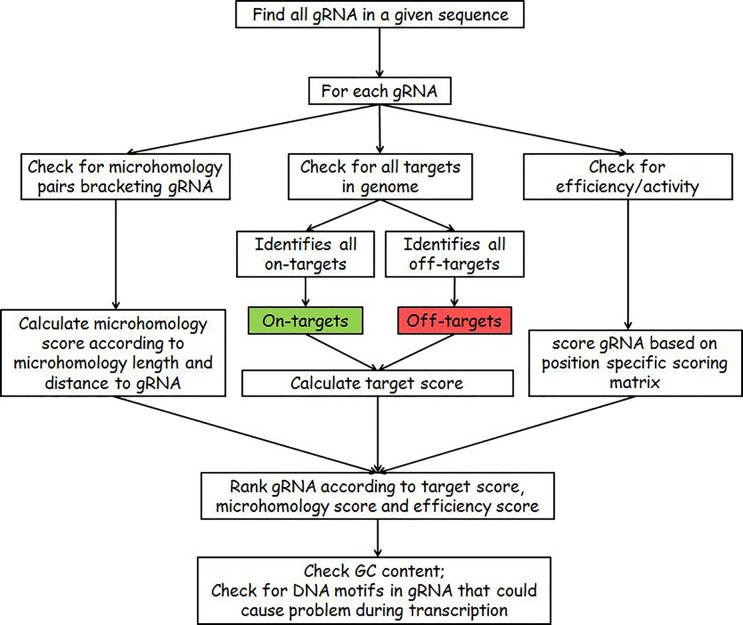
Example workflow of a non-batch job request.

EuPaGDT identifies all possible gRNAs in an input sequence, and then calculates a ranked list of those gRNAs based on (1) on-target and off-target hit(s) in the selected or uploaded pathogen genome, (2) predicted gRNA activity and (3) identified microhomology pairs flanking the gRNA targeted cut site. Fig. 1 illustrates a workflow for a non-batch job request.

**Fig. 2.**
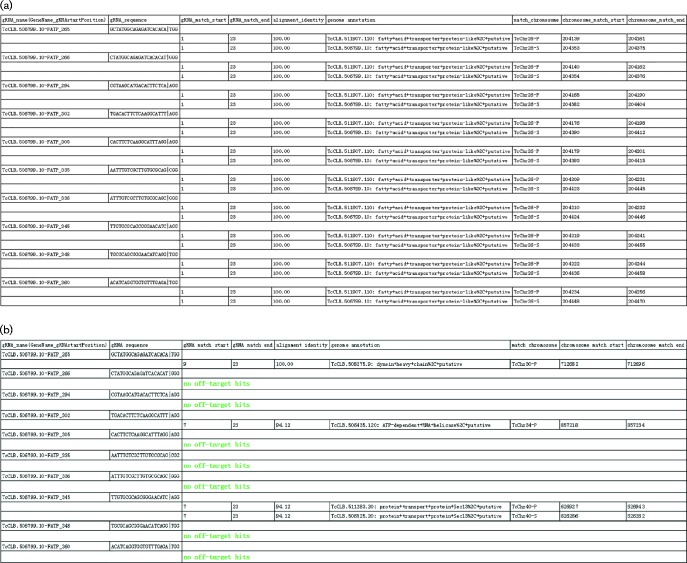
(a) Example output of genomic on-target hits and annotations for 10 gRNAs found in the TcFATP gene (gene id TcCLB.506799.10) in the *T. cruzi* CL Brener genome. (b) Example output of genomic off-target hits and annotations for 10 gRNAs found in the TcFATP gene in the *T. cruzi* CL Brener genome.

EuPaGDT can identify on-target hits in the genome for each gRNA, including those intended for editing multi-gene families. The on-target hit feature has the advantage of also identifying additional, sometimes unannotated copies of target genes. This feature is particularly important in incomplete and poorly annotated pathogen genomes, and/or in incompletely annotated gene family sets that can number in the thousands. To find all on-target genome-hits, the program compares the homology between sequences flanking the identified gRNA with all genome-target flanking sequences. If flanking homology regions meet the programmed identity criteria, then the genome-target will qualify as a potential on-target hit. Key parameters governing flanking homology comparison, such as sequence length and threshold for alignment identity and coverage, can be adjusted by the user to enable effective searching of on-targets in a spectrum of highly conserved to more divergent gene families. Identified similar sequences that do not meet these on-target criteria are automatically assigned to the off-target list. All genomic hits are annotated to aid the user in determining whether off-targets are within coding regions and to verify genomic loci annotations for each on-target site (Fig. 2).

A target score is assigned to each identified gRNA. The target score reflects how well a gRNA can target on-target sites while avoiding off-targets and is calculated as follows:

The first term of the target score equation is a fraction that reflects how well the current gRNA hits on-targets compared with the theoretical maximum target index. The maximum on-target index represents the maximum on-target number that gRNAs in a given input sequence can have; for example, a single-locus two-allele gene will have 2 as the ‘maximum on-target index’, and a gene with five copies in the genome would have 5 as the ‘maximum on-target index’. The first term of the equation has a maximum value of 1. The second term of the formula evaluates how well the current gRNA avoids hitting off-target sites. Briefly, if a gRNA has far more on-targets than off-targets, the second term would be a small fraction, which is deducted from the first term; in contrast, the second term will be >1 if a gRNA has more off-targets than on-targets, giving a negative target score. The target score equation as a whole works equally well for single-locus genes and large gene families.

To further assess the potential utility of each gRNA identified, we have adopted a gRNA activity prediction scoring matrix empirically determined by Doench *et al.* (2014). The scoring matrix scores gRNA based on the nucleotide composition of gRNA's 20 nt targeting sequence as well as 4 and 3 nt up- and downstream, respectively. Although the gRNA activity prediction scoring matrix is developed from data obtained in mammalian cells, we have found in *T. cruzi* that a higher activity score (>0.5) correlates strongly with successful gRNA function (*n*>10).

EuPaGDT is extremely flexible, allowing users to tune many parameters governing the design of gRNA and characterization of on-/off-targets. Recent engineering of CRISPR-Cas9 nucleases has identified several smaller Cas9 orthologues as well as ones with altered protospacer adjacent motif (PAM) preferences (Kleinstiver *et al.*, 2015). The expanding list of available PAMs greatly increases the number of potential gRNAs in a given nucleotide sequence, allowing researchers to edit genomes with higher precision. EuPaGDT by default is programmed to use ‘NGG’ as an on-target PAM and ‘NAG’ and ‘NGA’ as off-target PAMs for the most widely used Cas9 from *Streptococcus pyrogenes* (SpCas9). However, users can specify multiple, custom on-target PAMs. For example, using the standard International Union of Pure and Applied Chemistry code (Cornish-Bowden, 1985) to specify degenerate PAM(s) of 3–10 bp, users can input a degenerative PAM sequence ‘NNGRRT’ recognized by Cas9 from *Staphylococcus aureus* (SaCas9) plus a variant PAM sequence ‘NGA’ used by SpCas9, in addition to using the classical PAM ‘NGG’ for SpCas9. EuPaGDT also allows users to specify multiple off-target PAMs. For example, SpCas9 can recognize the ‘NGA’ PAM at a low level (Kleinstiver *et al.*, 2015); off-targets using such alternative PAMs can be evaluated individually and integrated into the selection process for gRNAs if desired.

Users can refer to the ‘table of on-/off-targets’ pages available from the main result output page for each job request to visually inspect each on-target/off-target and their corresponding PAMs, as well as the alignment of gRNA with the target sequence. EuPaGDT provides other customized parameters such as gRNA length [as shorter gRNAs are shown to have fewer off-targets (Fu *et al.*, 2014)] and on-target and off-target search parameters can be custom specified to accommodate users’ unique needs. For example: (1) searching for on-targets in a fast evolving multigene family might require relaxation of on-target searching criteria, lowering the ‘identity’ and ‘coverage’ parameter values in small decrements to determine if gRNA on-target numbers increase with a steady number of off-targets; and (2) selecting a shorter gRNA length might require a corresponding decrease in the ‘maximum number of mismatches’ allowed in the off-target parameter setting to ensure accurate off-target evaluation.

Microhomology-mediated end-joining (MMEJ) has been shown to repair double-stranded breaks (DSBs) generated by the CRISPR-Cas9 system in mammalian cells (Bae *et al.*, 2014; Wang *et al.*, 2013) and eukaryotic parasites (Peng *et al.*, 2015), often resulting in local sequence deletions which disrupt target genes. Analysing pairs of microhomology sequences that flank gRNA can help predict the size of MMEJ-mediated gene deletions. EuPaGDT identifies gRNA-flanking microhomology pairs of length 5–20 bp within 500 bp of each gRNA. Although MMEJ-mediated DSB repair is known to be involved in DSB repair in trypanosomes, the specific rules governing microhomology length and proximity to DSBs with respect to the efficiency of MMEJ DSB repair are not yet known (Glover *et al.*, 2011; Peng *et al.*, 2015). Therefore, EuPaGDT assigns each gRNA a score on a scale of 0–1 reflecting the length of microhomology pairs and their proximity to the gRNA-directed cut site, with a score of 1 for an ideal microhomology pair (>20 bp in length, and immediately flanking the gRNA cut site).

In addition to relying on MMEJ-induced deletions to mutate specific sequences, single-stranded oligonucleotides (ssODNs) bearing homology arms bracketing gRNA-guided DSBs can be used to introduce modifications at specific target positions by homology-directed repair (HDR; Wang *et al.*, 2013; Wu *et al.*, 2013; Yang *et al.*, 2013). Using such repair templates may also obviate undesired changes in the targeted gene (e.g. chromosome translocation or large-scale deletions; Brunet *et al.*, 2009; Cho *et al.*, 2014; Lee *et al.*, 2010). To aid researchers in designing oligonucleotide repair templates, EuPaGDT will by default generate an archetype ssODN sequence for each gRNA. Each ssODN repair donor has 30 bp of homology arms flanking gRNA's predicted DSB site and an 11 bp sequence consisting of three stop codons in three reading frames that will be inserted into the targeted sequence at the DSB site. Our default insertion site is at the DSB site (3 bp upstream of the PAM in SpCas9) because editing efficiency is highest when the desired nucleotide change is proximal to the DSB site (Bialk *et al.*, 2015; Yang *et al.*, 2013). Users can specify the length of the homology arms, and also enter the desired custom insertion-sequence in place of the default three-frame stop codon sequence. Further custom changes such as sequence deletion, nucleotide changes or insertion adjacent to the DSB site can be easily made at the user's discretion.

Additional quality control is performed by EuPaGDT to ensure that gRNA will be transcribed efficiently. EuPaGDT checks each identified gRNA for the presence of DNA motifs that may inhibit or terminate RNA polymerase transcription (Bogenhagen & Brown, 1981). EuPaGDT will also remind users to add a leading ‘G’ for efficient initiation of transcription when gRNA does not start with ‘G’.

EuPaGDT ranks all gRNAs found in an input sequence based on their total score, which is calculated by unweighted averaging of the respective target score, activity-prediction score and microhomology-pair scoring. Our repeated test runs using a variety of input gene sequences show that the ranking process performs well in placing gRNAs with more desirable traits closer to the top of the list. Users can rapidly identify desirable gRNAs using the ranked list and further choose gRNA of interest based on specific usage, for example targeting a specific region of the input sequence.

**Fig. 3.**
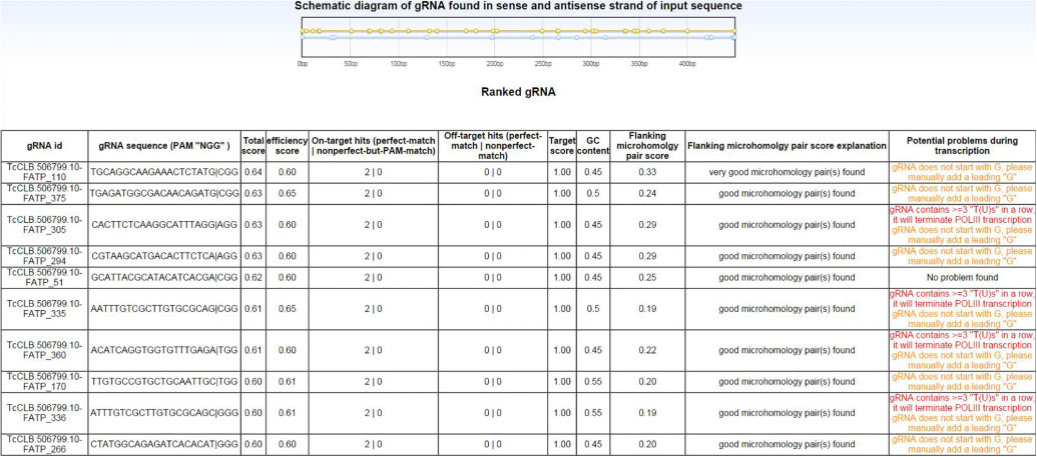
Example output of summary page of gRNA found in the TcFATP gene in the *T. cruzi* CL Brener genome (only the top 10 ranking gRNAs are shown).

**Fig. 4.**
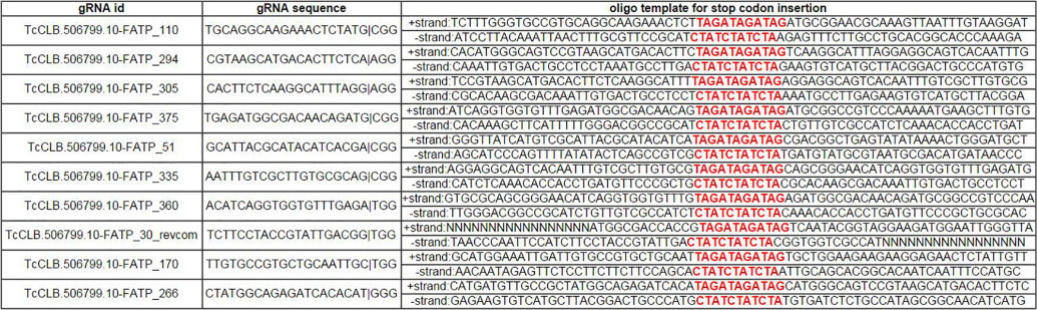
Example output of archetype ssODNs for the top 10 ranking gRNAs found in the TcFATP gene.

**Fig. 5.**
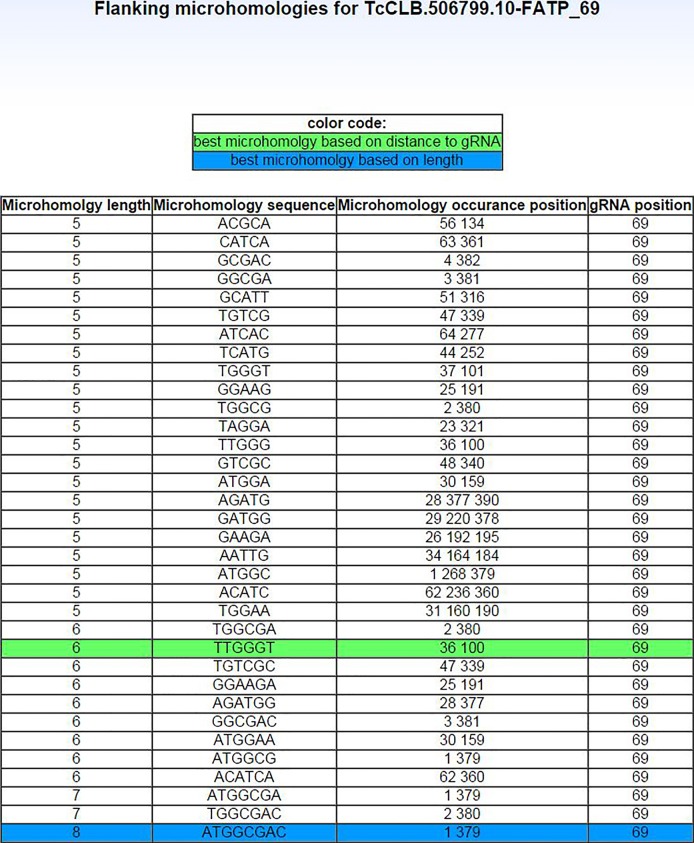
Example output of microhomology pairs found for a gRNA in the TcFATP gene.

For each request, EuPaGDT produces a summary page, including a schematic diagram showing each gRNA's position and strand in the input sequence, and a ranked list of such gRNAs along with concise results of each characterization step (Fig. 3). At a glance, users can easily grasp essential information relating to each gRNA. Additional detailed information [such as a summary of on-/off-targets (Fig. 2), archetype ssODNs (Fig. 4) and a summary of microhomology pairs (Fig. 5)] from each characterization step is also available to the user to allow for selection of gRNA suitable to the project.

EuPaGDT also features a ‘batch mode’, which can process a list of genes and return a user-defined number of top-ranking gRNAs for each gene. Additionally, a multi-threaded batch mode is available to process genome-scale gene lists for genome-wide screening projects. EuPaGDT is currently available for 25 eukaryotic genomes, covering major lineages of eukaryotic pathogens as well as several popular model organisms. Users can also upload custom genomes or request default genomes to be available in EuPaGDT.

Informed and well-scrutinized gRNA design is instrumental to successful CRISPR-Cas9-mediated genome editing or gene expression manipulation. Here we describe a gRNA design web tool, EuPaGDT, tailored to eukaryotic pathogen gRNA design. By characterizing potential gRNAs with an array of currently accepted principles, EuPaGDT can facilitate researchers of eukaryotic pathogens to arrive at proper gRNA design for CRISPR-Cas9 experiments and thus bring new understanding for these otherwise difficult to manipulate pathogens.

## Conclusion

We have developed EuPaGDT, a web-based tool, to assist the eukaryotic pathogen research community in designing gRNA and ssODN HDR templates for CRISPR-Cas9 experiments.
